# Development of Learning Media for the Elderly to Promote Child Health in the Community

**DOI:** 10.1155/2020/7252046

**Published:** 2020-10-29

**Authors:** Napalai Chaimaha, Supawadee Putthinoi, Suchitporn Lersilp

**Affiliations:** Department of Occupational Therapy, Faculty of Associated Medical Sciences, Chiang Mai University, Chiang Mai, Thailand

## Abstract

The purpose of this study was to develop learning media for the elderly to promote child health. The participants consisted of 172 elderly people aged 60 years and older in a suburban village of northern Thailand. This study consisted of a survey questionnaire and focus group discussions and was divided in to two phases: (1) exploring the needs of the elderly in the knowledge of child health and (2) developing learning media, so that the elderly can learn how to promote child health and evaluate its efficiency in the trial process. Results showed that the participants mostly preferred the topic of activities of daily living (ADL) in children. The learning media contributes two major contents: (a) knowledge of child development in five types of ADL performance, brushing, eating, dressing, bathing, and toileting, and (b) methods in teaching ADL skills in children. The digital contents in the learning media were proposed to experts for professional approval. Efficiency of the learning media was evaluated after the trial, when the participants expressed their opinion, and the users perceived that the learning media was effective, motivating, easy to use, and simple in the terms used, as well as appropriate in the sequence of contents and pictures and font, size, and color of the text.

## 1. Introduction

The elderly population in Thailand is increasing dramatically, and in 2018, it reached 11.7 million, or 17.6% of the total population [1]. The ability of elderly people to live independently as active aging with appropriate activities is mandatory. Active aging is a policy framework or guideline developed by the World Health Organization in promoting health. It consists of three components: health, security, and participation [2]. The elderly in a Thai suburban community have spent their time in varied activities such as rest and sleep, activities of daily living, work, education, leisure, and social participation [3]. As retired people, they live full time with their family and transform their major role from the past carrier to a caretaker for family members. Hence, family support and intergenerational relationships are very important. In the Thai community, a common role of the elderly in the family is raising their grandchildren, especially when the parents work outside the house and have little time for child care, and the number of children raised by grandparents has increased. Therefore, a major role of grandparents is to manage everything in the daily routine of their grandchildren [4]. Grandparents are happy to take care of their grandchildren, particularly those aged 1-3 years old or the first descendant of the family. They expect their children to be intelligent and have good quality of life [5]. However, the major problem for these grandparents is the lack of knowledge about childcare. As times change, their wisdom and skills of childcare from the past are obsolete, and they find their experience inappropriate for current trends [6]. Therefore, a solution for this issue is support, with knowledge or guidance on how to raise children through appropriate learning media. Grandparents with full-time responsibility for their grandchildren tend to benefit from their participation in multiple roles [7]. This also influences the development of children significantly in social, behavioral, and educational functioning [8]. Accordingly, a specific and useful resource or guideline, based on the needs and preferences of the elderly, is instrumental in caring for grandchildren. This concept was posted substantially in a previous research, in which grandparents identified that one of their service needs is education that focused on raising grandchildren (86.80%) [9]. In addition, the elderly currently show a growing use rate of information and communication technology (ICT) and technological devices such as computers, mobile phones, and the Internet. Technology has become a part of their everyday lives. They also understand how to use ICT and access digital media [10].

Occupational therapists (OTs) are contributors to health promotion and prevention in all age groups, which includes healthy lifestyles for all individuals and their families. OTs play an important role in encouraging and facilitating participation in meaningful occupations [11, 12]. From an occupational therapy (OT) perspective, occupation is defined as all the needs of the people and what they want to do [13]. If the elderly as grandparents want to spend their days caring for children, OTs can support or establish them to participate actively in this role by providing appropriate services and adaptive strategies [14]. Learning media is a learning strategy or learning tools provided by OTs for supporting the knowledge and skills in health promotion. This study, therefore, aimed at developing learning media for the elderly to promote child health in the community.

## 2. Methods

A developmental research design was used in developing the learning media for elderly people to promote child health in the community, as approved by the Research Ethics Committee of the Faculty of Associated Medical Sciences, Chiang Mai University, Thailand (AMSEC-62EX-013).

One hundred and seventy-two elderly people in Sannameng Village, San Sai district, Chiang Mai, Thailand, were selected as participants for this study by the purposive selection process with inclusion criteria: (a) aged 60 years or older, (b) grandparents who were raising or had raised their own children or grandchildren, and (c) voluntarily consented to participate in this study.

The instruments in this study were a survey questionnaire focusing on the topic of child health and focus group discussions. The questionnaire was designed and developed by the research team and then examined for content validity by five experts: two occupational therapy lecturers, two OTs, and a nurse, all of whom had worked in geriatric and community fields for at least five years. The Index of Item-Objective Congruence (IOC) was 0.97. The survey questionnaire consisted of demographic data of the participants and topics of promoting child health and was self-administered, with 15 items in a dichotomous scale checklist format, with yes/no questions.

This study was divided in to two phases: (1) exploring the need for knowledge among the elderly to promote child health and (2) developing the learning media for the elderly to promote child health. In these terms, the data were collected through a questionnaire and focus group discussions.

Phase 1: the survey on the need for knowledge of child health among the elderly was self-administered by the 172 participants. The data obtained from the questionnaires were rechecked for completeness and then analyzed by descriptive statistics.

Phase 2: development of the learning media for the elderly consisted of learning how to promote child health and evaluating efficiency of the media in the trial process. The researchers developed the media in this phase on the basis of information derived from the first phase. This phase comprised three steps as follows:

1. Designing contents of the media. The researchers designed contents of the media by collecting in-depth data of the most preferable topic through four sessions of focus group discussions with 36 elderly people (36 available elderly from the 172 participants volunteered to participate in four focus group discussions). The researchers divided the participants into 5 focus groups (7-8 people per group) and met them every month for 4 months. The first focus group discussion aimed at obtaining details of the contents required by the elderly as a resource for starting development of the learning media through a question guide. After that, the researchers gathered more information by reviewing related literature in order to complete the contents. More elaborate details of the learning media design were discussed in the second discussion. After the first draft of the learning media was developed, the focus group members analyzed and discussed the overall image and contents in the third and fourth focus group discussions
2. Developing the learning media. The data from the first draft were then transformed to digital contents on a web base. Face validity assessment was conducted with five experts to determine the appropriateness of overall image and contents of the learning media
3. Usability testing. From the 172 participants, efficiency of the learning media was assessed by 58 elderly people who were volunteers and available for using the program. They conducted a trial session for 30 minutes before evaluating efficiency of the learning media by using a questionnaire, which comprised 8 items that were classified into three types: agree, neutral, and disagree.

## 3. Results

**Figure 1:**
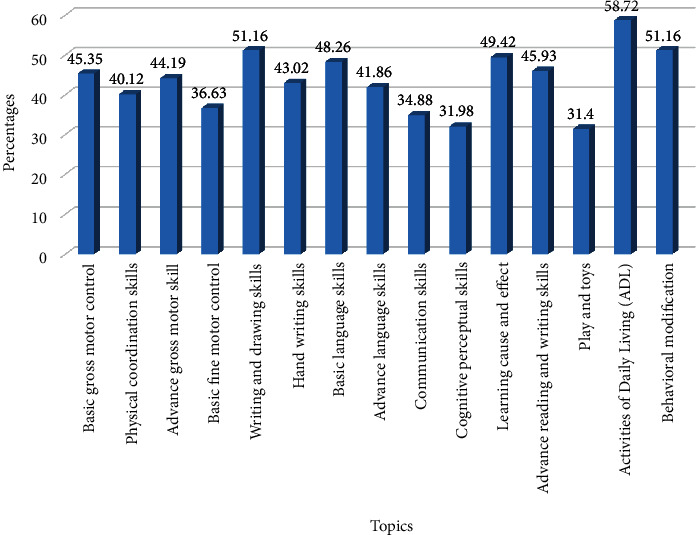
Percentages of topics on child health in the learning media for the elderly (*n* = 172).

**Figure 2:**
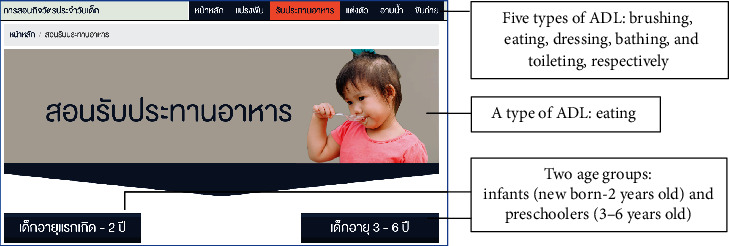
Two age groups.

**Figure 3:**
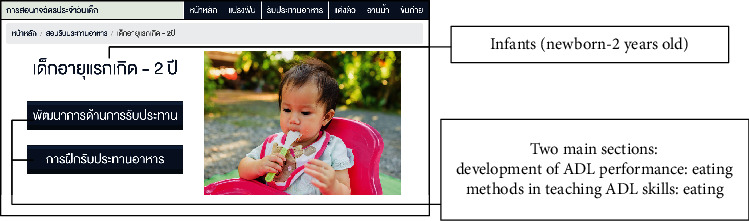
Two main sections of the knowledge media.

Findings from the questionnaire reflected the needs for the elderly to gain knowledge of the topics in the learning media in order to promote child health. The 172 participants comprised 35 males (20.35%) and 137 females (79.65%), with the largest number aged between 65 and 69 years old (36.63%), and half of the total being married (54.70%). Most of them graduated from primary school, the national basic education (69.77%). Characteristics of the participants are shown in Table 1. The percentages of topics needed in the learning media for the elderly to promote child health are illustrated in Figure 1. The learning media for the elderly to promote activities of daily living (ADL) in digital contents for the children is illustrated in Figures 2 and 3.

### 3.1. Development of the Learning Media for the Elderly to Promote ADL in Children

**Figure 4:**
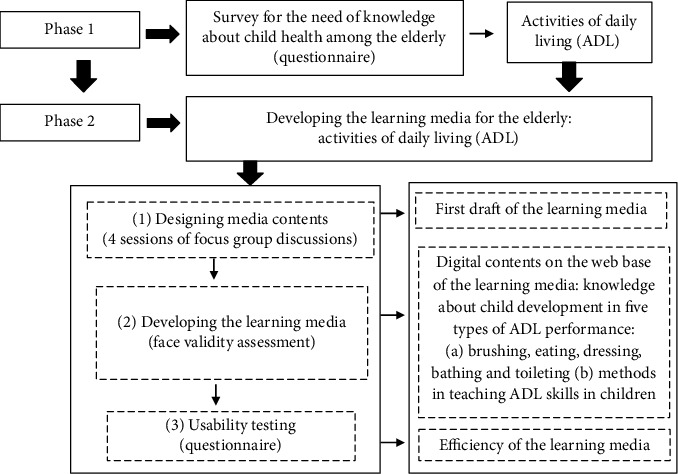
Process for developing the learning media.

The learning media was designed and divided into two phases. Researchers in the first phase surveyed the need for knowledge about child health among the elderly. Then, the learning media was developed in the second phase. The process of developing the learning media progressed from the starting point in phase 1 to its efficiency assessment in phase 2, as shown in Figure 4. The results of phase 1 indicated that ADL (58.72%) was the most common topic required by the elderly to create the learning media for promoting child health. The second most common topics required were behavioral modification and writing and drawing skills, which shared the same score (51.16%) (Figure 1).

After topics in the learning media had been selected, the researchers continued to develop phase 2, in which they developed the learning media on the topic of ADL in children. The first draft of the learning media was designed and developed through four focus group discussions, the first of which displayed the domain and detail of contents required by the elderly as a resource to start development through a question guide. Most of the elderly needed to know two themes of information about ADL in children aged between newborn and 6 years old that comprised (a) development of knowledge on child development in five types of ADL performance, brushing, eating, dressing, bathing, and toileting, and (b) methods in teaching ADL skills in children. After that, the researchers gathered more information in order to complete the contents by reviewing related literature. The second discussion talked about more elaborate design details of the learning media. Most of the participants in this session expressed their opinions on the contents and aspect of the pictures and font, size, and color of the text. They preferred an enlarged font size, contrasting color of background to text, more use of pictures than text, and natural photographs of children. In the third and fourth focus group discussions, the participants analyzed and discussed the overall image and contents after the first draft of the learning media had been developed.

After designing the contents of the learning media through the focus group discussions, the digital contents that promote ADL in children for the elderly were designed in the form of a digital system installed on an Internet server. The contents of knowledge on the media comprised (a) knowledge about development in the five types of ADL performance, brushing, eating, dressing, bathing, and toileting, and (b) methods to teach ADL skills in children aged between newborn and 6 years old. Face validity assessment was conducted with five experts in order to determine the appropriateness of the overall image and contents in the learning media. The experts made suggestions to increase the font size, adjust the contrast of background to font, and add more pictures in the contents. After that, knowledge of the learning media was adapted according to guidance of the experts. The effectiveness of the learning media was assessed after a trial session by using the usability testing questionnaire (Table 2). The samples of the pages in the learning media are presented in Figures 2 and 3.

The learning media for the elderly to promote ADL in children in the community was designed as follows; the home page of the media shows its topic in the middle of the page. The five types of ADL, brushing, eating, dressing, bathing and toileting, are presented as icons at the top of each page, and their pictures are illustrated at the bottom. Users are able to access their ADL of interest by clicking on the appropriate icon. Then, the users can choose the age range of the children: e.g., infants (newborn-2 year olds) and preschoolers (3-6 years old), as shown in Figure 2. Two main sections in the learning media are shown in Figure 3, where the development of ADL performance and methods in teaching ADL skills in children can be seen. This page appears after users click on their chosen age range. In-depth details in each section may be approached by clicking on the icons.

Evaluation on efficiency of the learning media was assessed after the trial, when the participants gave their opinion. Results from the perspective of 58 users indicated that the design of the learning media was effective in stimulation (100%), easy to use for the first time (91.38%), easily presented (98.28%), and simple to describe (100%), as well as having appropriate sequence of content (96.56%) and pictures (98.28%) and font, size (96.55%), and color (94.83%) of the text. Percentages of feedback from the elderly after the trial are presented in Table 2.

## 4. Discussion

This research started by exploring the need of knowledge for the elderly on promoting child health. The results of this study showed that most of the participants preferred the topic of ADL, specifically basic ADL or daily self-care activities (brushing, eating, dressing, bathing, and toileting). This was similar to previous studies about the important roles of the elderly in the basic care and activities of their grandchildren, including eating, sleeping, and playing with them, which varied depending on the age of the children and their family needs and structure [5, 15, 16]. Grandparents who provide care for their grandchildren meet burdens but also benefit from their roles, such as feeling more useful or valuable [17, 18]. Therefore, increased understanding of the special needs and problems of the elderly in this situation enhances the effectiveness of their role, which closely relates to child health and family relationships [19]. Similar to previous studies [19], this study also considered the needs of the elderly and focused on the specific topics required. Then, the learning media developed the resource or information that met the needs of the elderly and provided suggestions for establishing caregiving roles of the elderly as grandparents in promoting ADL to the children. The learning media contained resources that focused on providing information on developing the five types of ADL performance: brushing, eating, dressing, bathing, and toileting. It also provided methods in teaching ADL skills in children aged between newborn and 6 years old, which is beneficial for both the grandparent as the caregiver and the grandchild as the care receiver. Providing specific information for the elderly who raise their grandchildren, such as childhood development and coping strategies on related issues, leads to positive outcomes [16]. Users of the learning media were able to choose information about child development in the five aspects of ADL performance as well as the methods used in teaching ADL skills in children, such as development of eating and brushing in preschoolers, methods of teaching infants to eat, and methods in teaching preschoolers to dress themselves. The users also could use backtracking on each content by clicking on the icons presented on each page of the learning media.

The research team developed the learning media based on the needs of the elderly, and the data were transformed to digital content in web base design. This is in line with the present trend of the elderly learning and showing more use of information and communication technology (ICT) than in the past, such as using computers, mobile phones, databases, and the Internet [10]. In addition, media technologies are designed to benefit and support an active life [16]. In this research, the learning media was created as assistive technology to promote elderly people in the duty of raising their grandchildren. After the trial in this study, the perspective of users indicated that design of the learning media was effective in its motivation, easy to use for the first time, easily presented, and simple to describe, as well as having the appropriate sequence of contents and pictures and font, size, and color of the text. This completeness helped in identifying efficiency of the learning media when used by the target users—the elderly in the community. This tool helped the elderly, as grandparents, to learn about caring for or raising their grandchildren in the aspects of development in the five types of ADL performance and methods in teaching ADL skills in children aged between newborn and 6 years old. Not only grandparents but also grandchildren benefited from this innovative media, as the caregiver and care receiver, respectively. Furthermore, the learning media was a resource guide in promoting the ADL of children and assisting inexperienced parents or caregivers who provide care to children.

The sample of participants from only one area of northern Thailand was a limitation of this study, and it cannot be generalized for all of the Thai elderly population. Further studies should include participants in all areas of Thailand. In addition, the needs of the elderly were explored by the quantitative method that focused on motor, language, and cognitive areas. This method limited the scope of needs. Therefore, further study should use various methods of data collection, especially the qualitative method. Moreover, the scope of needs might be considered other aspects such as mental health, and social and emotional development, thus leading to improved learning media that are able to enhance the normal development of children together with their grandparents' perspectives.

## 5. Conclusion

The learning media was developed for the elderly in this study to promote child health as an innovative outcome. It comprised two main contents including development in five types of ADL performance, brushing, eating, dressing, bathing, and toileting, and methods in teaching ADL skills in children. Moreover, after the trial, the perspective of the users indicated that the learning media was effective and beneficial for the community, specifically for the elderly, who were active aging in raising their grandchildren, and also for the children, who were trained appropriately in ADL.

## Figures and Tables

**Table 1:** Characteristics of the participants (*n* = 172).

Characteristics	*n*	(%)
Gender		
Males	35	20.35
Females	137	79.65
Age (years)		
60-64	53	30.81
65-69	63	36.63
70-74	27	15.70
75-79	20	11.63
>79	8	4.65
Marital status		
Single	5	2.90
Married	94	54.70
Widowed	73	42.40
Education		
Uneducated	4	2.33
Primary school	120	69.77
Junior high school	16	9.30
Senior high school	25	14.53
Bachelor's degree	6	3.49

**Table 2:** Efficiency of the learning media (*n* = 58).

Opinion on efficiency	Agree (%)	Neutral (%)	Disagree (%)
Effective in stimulation	100.00	0	0
Easy to use for the first time	91.38	8.62	0
Easily presented	98.28	0	1.72
Simple to describe	100.00	0	0
Having appropriate sequence of content	96.56	0	3.44
Having appropriate font and size of text	96.56	1.72	1.72
Having appropriate color of text	94.84	1.72	3.44
Having appropriate pictures	98.28	0	1.72

## Data Availability

The data used to support the findings of this study are available from the corresponding author upon request.
